# Metabolic rates in the squareback marsh crab *Armases cinereum*

**DOI:** 10.1002/ece3.9665

**Published:** 2022-12-28

**Authors:** Nanette Smith, Lars Anderson, Laura S. Fletcher, Carter K. Stancil, Blaine D. Griffen

**Affiliations:** ^1^ Department of Biology Brigham Young University Provo Utah USA

**Keywords:** ecological energetics, energy budget, energy subsidy, field metabolic rate, salt marsh

## Abstract

Metabolic rate is a basic individual metric that extends beyond the individual to link the ecology of populations, communities, and ecosystems via its role as a central component of energy budgets. Metabolic rates are often measured indirectly by quantifying respiration under simplified, standard laboratory conditions. This approach limits the application of these measurements to a small range of conditions that commonly do not reflect natural field conditions. We measured metabolic rates of the squareback marsh crab *Armases cinereum* under field conditions. Previous work highlights that movement of individual *A. cinereum*, especially females, provides a potential spatial subsidy of energy, as individuals consume foods in salt marshes and then transfer the resulting energy to upland forest ecosystems. We show that metabolic rate increases with size for both males and females and that metabolic rates are influenced by temperature and by whether females are vitellogenic. The metabolic rates that we measured more closely approximate field metabolic rates than standard metabolic rates and demonstrate that individual crabs experience high energy expenditures, reducing the amount of energy that may be transferred as a subsidy between marsh and forest as a result of the daily movements of individual crabs. Our measurements are therefore also a key component for the construction of an energy budget for this species.

## Graphical abstract

We show that the flatback marsh crab *Armases cinereum* has relatively high rates of energy use. This likely limits the ability of this species to act as a vector for energy subsidies between salt marsh and forest habitats, as has previously been hypothesized.

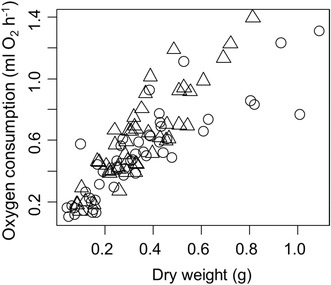

## 1 INTRODUCTION

Metabolic rate has been described as “the most fundamental biological rate” as it links individual organisms to the ecology of populations, communities, and ecosystems (Savage et al., 2004). It is central to many biological and ecological processes, and many metabolic theories exist to explain these connections (Kearney & White, 2012; Van der Meer, 2006). Metabolic rate has been used to predict individual growth (West et al., 2001), food‐web structure (Brown & Gillooly, 2003), and global patterns of abundance (Kaspari, 2004), among other things. While historically studies have primarily measured metabolic rates under constrained laboratory conditions, more recent work has focused on ecological energetics and the measurement of metabolism under more natural conditions experienced by animals on a daily basis (Tomlinson et al., 2014).

Two of the most prominent theories regarding the measurement of metabolic rate have been proposed by Kooijman (1986) and Brown et al. (2004). Kooijman introduced dynamic energy budget (DEB) theory (Kooijman, 1986) that allows for the analysis of individual processes such as ingestion, assimilation, respiration, growth, and reproduction within a single framework (Van der Meer, 2006). DEBs produce quantitative measurements of the rates at which an organism assimilates and utilizes energy. According to the DEB theory, the rate of energy assimilation and usage is a function of the organism's state and its environment. The state of the organism is determined by individual characteristics such as size, amount of reserves, and age, while the environment is characterized by temperature and food density (Van der Meer, 2006). Brown et al. (2004) later presented the Metabolic Theory of Ecology (MTE). This theory focuses on metabolic rate as a function of body size and temperature. Both theories rely on the law of conservation of energy to build energy budgets for an individual organism that can be scaled beyond the individual to populations and communities (Van der Meer, 2006).

Concepts embedded in DEB and MTE can be formally integrated using energy budgets, tools that quantify the amount of energy taken in from the environment, and the amount of energy expended for respiration, development, growth, reproduction, and maintenance. In the simplest case, the energy intake is derived from the immediate surrounding environment. But some organisms instead rely on spatial subsidies, energy from a non‐local source, to meet their energy demands. In order to calculate an energy budget and consider the influence of spatial subsidies, the most fundamental starting point, the metabolic rate of the organism, must be known.

An interesting example of a mobile organism that relies on spatial subsidies by consuming food in different habitats is the squareback marsh crab *Armases cinereum*. This species is found in salt marshes and the surrounding upland forest habitat (Hübneṙ et al., 2015; Seiple, 1979) from the Chesapeake Bay south to the tip of Florida in the Atlantic and around the Gulf of Mexico down to Veracruz, Mexico. *A. cinereum* is a highly mobile crustacean (Seiple & Mueller, 1992) that occupies both of these habitats and acts as a link between the marsh and forest via bi‐directional movement. The species serves an important ecological role in mediating energy fluxes between trophic levels and along the marine–terrestrial ecotone (Treplin et al., 2013).

While this omnivorous crustacean inhabits both the marsh and the upland habitat, there are sex‐specific differences in habitat use. Thus, the amount of energy, matter, and nutrients that each crab transfers is dependent on their sex because sexual dimorphism influences diet choice and habitat choice (Hübneṙ et al., 2015). Gravid females prefer to occupy the marsh, but exhibit a large activity range both within the marsh and in the forest (Hübneṙ et al., 2015). They feed preferentially in the marsh and therefore may also act as a key biotic landward vector of spatial subsidies as the energy they consume in the marsh is transferred to the upland forest through their movements (Hübneṙ et al., 2015). In contrast to females, male *A. cinereum* prefer to occupy the forest habitat and exhibit a more restricted range, which largely excludes the marsh (Hübneṙ et al., 2015). Males have larger bodies and claws than females, which enables them to consume a larger amount and a more diverse range of prey species (Buck et al., 2003). This sex‐specific habitat use and energy transference likely means that the females of this species are the vectors of spatial subsidies as opposed to the males.

Organisms with highly mobile behaviors, like *A. cinereum*, may rely on and contribute to multiple food webs (Kiskaddon, 2016). Due to their broadly omnivorous diet, *A. cinereum* feeds at multiple trophic levels. For example, they have been observed to eat fresh leaves of various plants, partially decomposed leaves, insects, aphids, snails, other crabs, marsh sediments, and mammal feces (Ho & Pennings, 2008; Kiskaddon, 2016; Zimmer et al., 2004). Their mixed diet suggests that different food types may provide complementary nutritional resources leading to increased growth (Buck et al., 2003). These characteristics of *A. cinereum* may ideally be integrated using an energy budget that connects its energy use and movement across marsh and forest habitats.

Within saltmarsh habitats, energy budgets have been determined for grasshoppers (Smalley, 1960), fiddler crabs (Cammen et al., 1980; Shanholtzer, 1973), wading birds (Christy et al., 1981), and transient fish (Weinstein et al., 2000), but there is not a current energy budget for *A. cinereum*. Several components required for a formal energy budget have been quantified for *A. cinereum*, including consumption (Kiskaddon, 2016), growth (Buck et al., 2003), and reproduction (Figueiredo et al., 2008). The remaining two factors, assimilation and metabolic rate, have not been as thoroughly examined. Two prior studies measured respiration in *A. cinereum* (Full et al., 1985; Teal, 1959). In both of these studies, however, the data presented were not sufficient to understand how metabolism changes with body size, temperature, or other factors that are needed for use in an energy budget (i.e., the ecological energetics, sensu Tomlinson et al., 2014). Here, we seek to fill this gap and therefore to make a step towards the development of an energy budget for this species.

We measured the respiration rates of *A. cinereum* in air in outdoor laboratory conditions similar to what the crabs experience on a day‐to‐day basis. We hypothesized that the metabolic rate will increase with body size and temperature, in accordance with the MTE, and will be higher in males than in females. We further hypothesized that metabolic rates of gravid females will be higher than nongravid females and that metabolism will be higher in injured (crab missing limbs) than in noninjured individuals.

## 2 METHODS

We measured the metabolic rate of *A. cinereum* during July 2021. We collected 107 individuals on the grounds of Hobcaw Barony in Georgetown, South Carolina at 33°20′5.33″N 79°11′38.81″W over a three‐day period. We collected crabs along a marsh access road that divides the marsh from the upland forest. Upon collection, we contained the crabs in an enclosed cooler at ambient temperature until we measured their metabolic rates that same day.

### 2.1 Metabolic rate measurements

We conducted the experiments in a screened in outdoor laboratory at the field station for the Baruch Institute for Coastal and Marine Science (<2 km from the collection site). We collected metabolic data on gravid females, non‐gravid adult females, and adult males. Injury via limb loss is a common phenomenon in crabs with important implications for dietary intake and energy expenditure (Juanes & Smith, 1995). We therefore included crabs with existing injuries; however, if a crab dropped limbs while being collected or held before measurements, it was released and not used in the study as their metabolic rates may have been abnormal due to immediate stress.

We rinsed the crabs in saltwater and placed each individually into their own clear 150‐ml numbered polypropylene syringe. We previously sealed the tips of the syringes with silicone and drilled a hole into the side of each that was approximately 8 mm in diameter to serve as a port for sample extraction. The hole allowed free exchange of oxygen with the atmosphere until it was closed when the measurements began. Before sealing the holes in the syringes, we measured relative humidity, temperature, and barometric pressure with a digital anemometer (BTMeter 100‐AAP). We measured the ambient temperature approx. every 10 min throughout the trials to obtain an average temperature reading over the course of each trial. Each crab was only used in one trial. Crabs were given a 5‐min acclimation period after introduction into a syringe. A trial period was initiated when the hole was covered with a septum. The septa (model #001620; Bridge Analyzers Incorporated) are designed for use with headspace oxygen analyzers.

Depending on the size of the crab, we adjusted the syringe volumes from 35 to 110 ml. We used larger volumes for larger crabs to avoid hypoxia. In addition to varying the volume of experimental chambers, we also varied the duration of trials for individual crabs based on body size (20–90 min), with the experimental duration inversely related to crab body size, again to limit the risk of hypoxic conditions for larger crabs that had higher respiration rates. We chose the air volume and duration of the trial based on a visual assessment of the size of the crab, and we accounted for variable quantities for these two metrics in the calculation of metabolic rate for each crab (described below). As a result of these two procedures, oxygen levels in experimental chambers always remained well above levels that cause problems due to anoxia for air‐breathing crustaceans (Schmitz & Harrison, 2004). Specifically, minimum final oxygen level encountered was 19.72%, with mean final levels at 20.20% ± 0.13%. Similarly, carbon dioxide levels in experimental chambers always remained well below hypercapnia levels that cause problems for crabs (Burnett, 1997). Specifically, the maximum final carbon dioxide level encountered was 0.5%, with mean final levels at 0.29 ± 0.10%.

Immediately after covering the hole with the septum, creating a closed chamber, we started a timer to mark the beginning of a trial. We initially also recorded the movement of each crab every minute throughout the duration of the trial as either active or inactive. If the crab was walking or moving one or more of its legs, it was considered active. If the crab was completely still, it was categorized as inactive. However, activity during the trials was very rare, and when it did occur, it usually consisted of only very slow movements of one or two limbs. We therefore discontinued these measurements after the first half of the trials. Throughout the experimental period, we limited sudden movements to reduce visual cues that could startle the crabs and thus change crab metabolic rates.

At the end of each trial, we used a multigas oxygen probe from Forensics Detectors™, model # FD‐600 (0.01% resolution), to measure the final oxygen content in the syringe. We did this by inserting a needle through the septum and using the integrated instrument pump to draw air out of the chamber at a rate of 0.5 L/min. If there was no change to the oxygen reading at the end of the trial compared to the initial atmospheric concentration of 20.94% oxygen, then the crab was released and the data from that crab was discarded. No changes to the readings at times occurred because the septum came loose due to humidity, allowing gas exchange with the ambient air. These instances were rare and were readily apparent. The septa used were designed for this application and in all other instances provided an airtight seal. In a few instances, oxygen concentrations did not change measurably because allotted chamber volume that was too large or the time interval used was too short to enable detectable oxygen consumption. Crabs whose oxygen levels changed measurably were immediately placed in individually numbered bags and placed in a freezer at −20°C for later dissections.

### 2.2 Crab dissection

We transferred frozen crabs to Brigham Young University on dry ice and then placed them in a freezer at −80°C until dissection. Prior to dissection, we thawed crabs to room temperature in tap water, while still in their individual bags. We then measured the crabs' carapace width as well as the number of limbs lost and/or regenerating (based on the presence of limb buds). We determined whether females were gravid and/or vitellogenic using dorsal dissection and visual inspection of the ovary. We then placed each crab in a preweighed tin boat in a drying oven set to 60°C and dried them to constant weight. Crabs were weighed to the nearest 0.01 mg using a Mettler Toledo DualRange scale (model number XS205). After crabs had been dried and weighed, we additionally visually inspected the guts to assess whether they contained food and therefore whether crabs had been engaged in digestion during the metabolic rate measurements. This assessment was complicated by the fact that the gut becomes somewhat opaque upon drying. Three researchers independently assessed whether each gut contained food, and there was considerable variation between their assessments. Therefore, while we qualitatively report on this factor, we did not include this variable in our statistical analyses because of the lack of confidence about the gut fullness of any given crab.

### 2.3 Metabolic rate calculation and statistical analysis

We used equation 4.4 from Lighton (2018) to calculate the metabolic rate of the crabs, as follows:

$$\mathrm{Vol}O_{2}=\frac{\left[V\left(F_{i}O_{2}-F_{e}O_{2}\left(\mathrm{Vol}H_{2}O\right)\right)\right]}{\left[1-F_{e}O_{2}\left(\mathrm{RQ}\right)\right]}$$

The volume of the chamber (*V*) must exclude the volume of the crab. We determined the crab volume post hoc using the wet weight which was converted from the dry weight measurements we obtained. We used the conversion for crustaceans that dry weight is approximately 0.26× the wet weight (Ricciardi & Bourget, 1998). We then divided the calculated wet weight by the assumed density of crabs in order to get the volume. We used 1.1 g/cm^3^ as the density because the density of water is 1.0 g/cm^3^ and crabs generally sink in water. While this rough method of estimating crab volume admittedly has error, the small size of these crabs relative to the experimental chamber volume ensured that relatively minor errors were introduced into the overall calculations (crab volume was 1.5 ± 0.5% the volume of the experimental chambers).

We measured both the fractional concentrations of oxygen at the beginning of the trial, F_i_O_2_ (i.e., 0.2094, atmospheric concentration of oxygen) and the fractional concentrations at the end of the trial, F_e_O_2_, using the multigas oxygen probe. We included a small volume of water (<1 ml) in the experimental chambers to ensure saturation so that there was no change in water vapor during a trial. We therefore set the change in water vapor (VolH_2_O) to zero. We used a value of 0.85 for the respiratory quotient (RQ). This was based on the literature that classifies *A. cinereum* as an omnivore. While this value is likely accurate for this species, a value of 0.85 is also a middle‐of‐the road value (values generally range from 0.7 to 1.0) that minimizes the error associated with uncertainty about the quality of the food being metabolized during the experimental period. Based on a value of 0.85, the maximal error in metabolic calculations is roughly 3% (Vleck, 1987). Finally, we converted metabolic rates to standard temperature and pressure using the following equation from Lighton (2018):

$$\text{Corrected}\;\mathrm{\mu l}=\frac{\left(\mathrm{\mu l}\times \mathrm{BP}\times 273.15\right)}{\left(101.325\times T\right)},$$

where BP is the barometric pressure in kilopascals and *T* is the temperature in Kelvin.

We first analyzed male and female crabs together in order to compare scaling of metabolic rate with body mass for the two sexes. Following this, we then analyzed male and female crabs separately because some variables (vitellogenic and gravid) related only to female crabs. For all analyses, we used linear models in R version 4.1.2 (R Core Team, 2019). For each of these analyses, we used metabolic rate (ml O_2_ consumed per hour) as the response variable. As predictor variables in the models for the separate sexes, we used dry mass (g), number of limbs missing, number of limbs regenerating, average temperature (°C) during the trial, whether a crab was vitellogenic (for females), and whether a crab was gravid (for females). Each of these predictor variables is expected to influence energy expenditure, and reasonable arguments can be made for possible interactions between any of them. We therefore had no a priori expectations as to what interactions should be ecologically relevant. Consequently, we first fit a full model that included all these main effects and all interactions. We then used the step function in R to determine the best‐fitting model based on AIC using backward stepwise regression. We report only the best‐fitting models here.

## 3 RESULTS

Metabolic rates were influenced by multiple ecological factors that we did not attempt to control. One of these was natural limb loss and regeneration. Overall, 21 of the sampled males were missing limbs, with 13 of them regenerating lost limbs, while just 10 of the sampled females were injured, and five regenerating lost limbs. Additionally, metabolic rates may have been influenced by digestive processes for crabs with food in their guts. However, there was low repeatability in the estimates by three researchers that independently assessed the presence of food in the gut of each dried crab. Overall, they estimated that 61%, 70%, and 74% of crabs had food in their guts at the time that metabolic rates were measured. We found that activity was consistently low throughout the trials. The average trial duration was 47 min across all crabs, and for the subset of crabs on which activity was measured, males crabs were active during only 2.2 ± 4.5 min on average, while females were active during 3.4 ± 6.6 minutes on average. In addition to the limited time that crabs were active, the level of activity was also minimal, usually reflecting only slow movement of one or two limbs.

**FIGURE 1:**
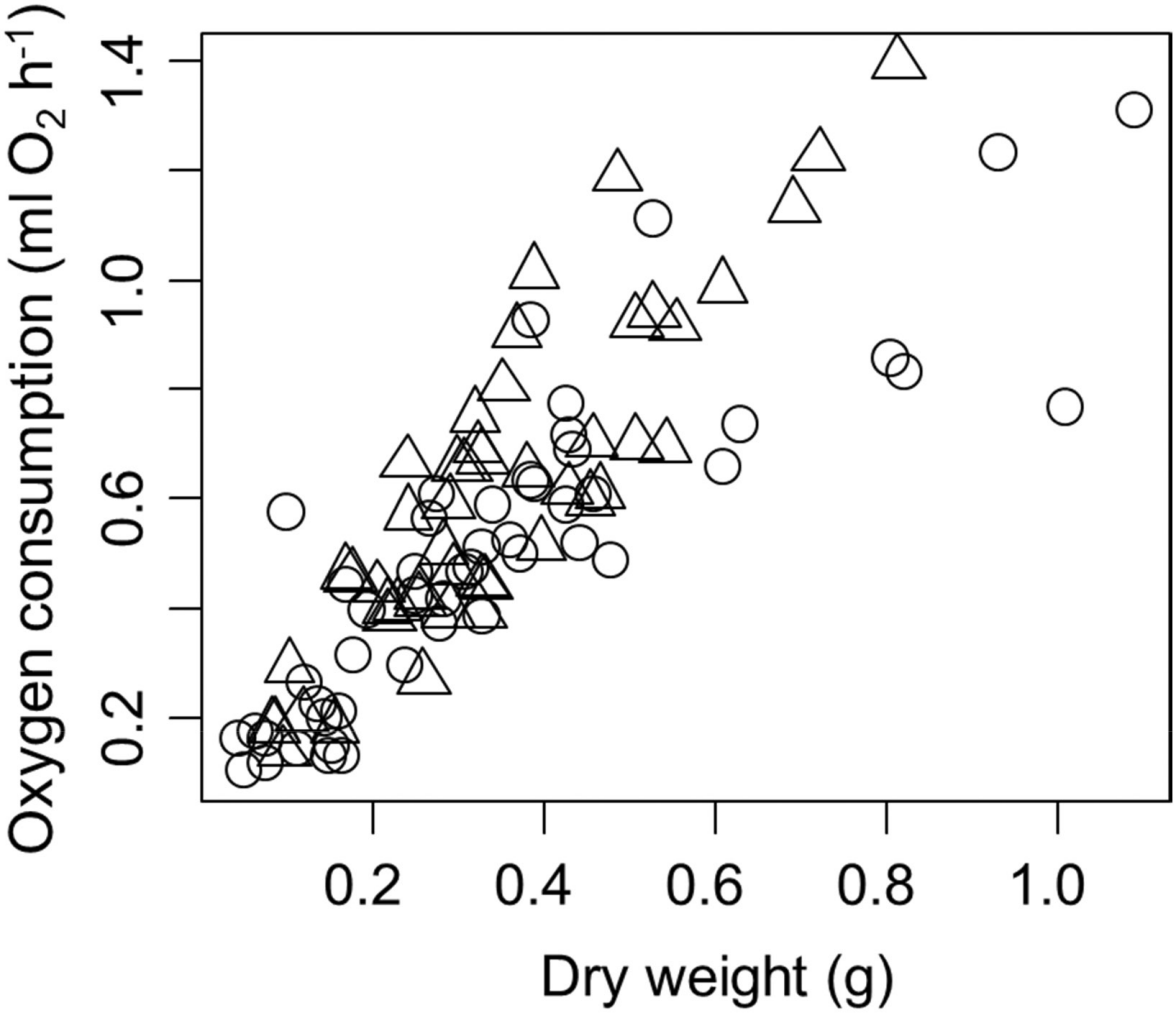
Metabolic rate of male (circles) and female (triangles) *Armases cinereum* as a function of dry body mass.

We found that metabolic rate increased with the dry weight of the crab (*t* = 12.21, *p* < .0001, Figure 1), but that the scaling with body weight differed for male and female crabs (weight by sex interaction term *t* = −3.58, *p* = .0005, Figure 1).

**FIGURE 2:**
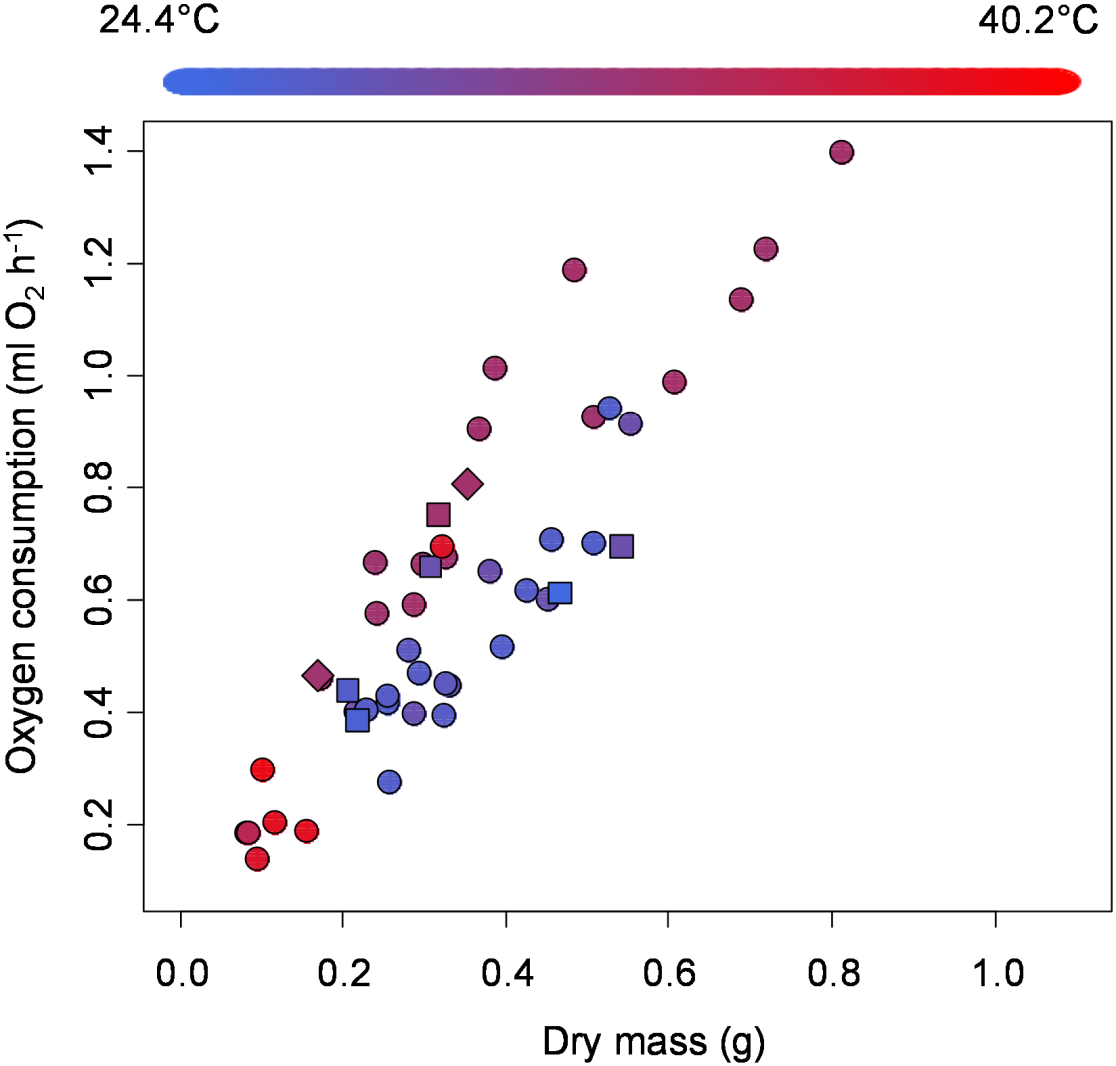
Metabolic rate of female *Armases cinereum* as a function of dry body mass (*x*‐axis), the number of limbs that were missing (circle = 0, square = 1, diamond = 2), and average temperature during experimental trials (symbol colors using the scale shown along the top of the graph).

**FIGURE 3:**
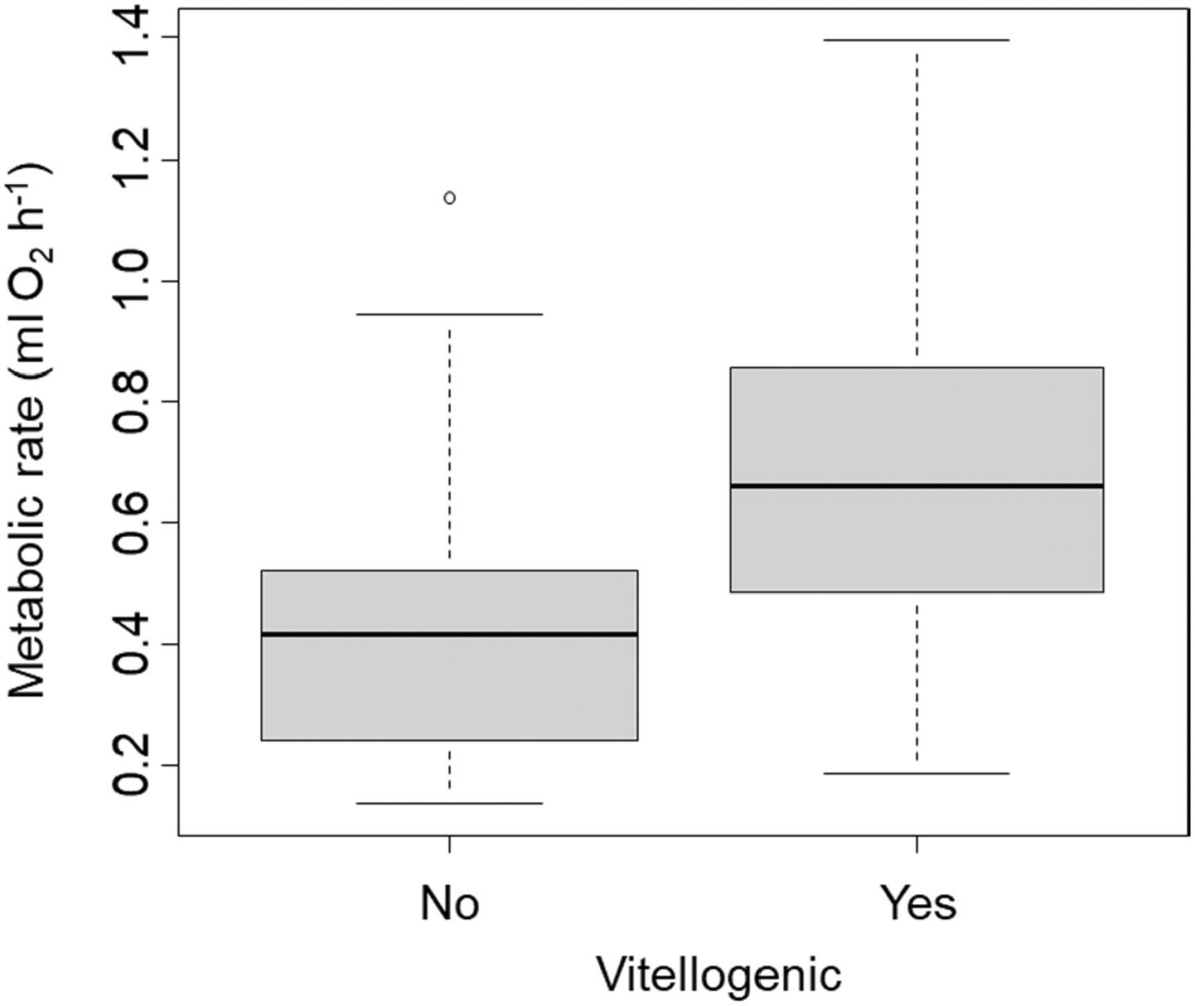
Metabolic rate of female *Armases cinereum* that either were or were not vitellogenic at the time of the trials. Thick center lines show median values, the boxes encompass the interquartile range, whiskers show 1.5× the interquartile range, and the circle is a single outlier that falls outside that range.

For female crabs, we found that metabolic rates increased with body mass (*t* = 9.30, *p* < .0001, Figure 2), with the average temperature during trials (*t* = 3.21, *p* = .004, Figure 2), with vitellogenesis (*t* = 2.16, *p* = .042, Figure 3), and with the number of limbs that were missing (*t* = 2.87, *p* = .009, Figure 2). We also found that metabolic rate was influenced by interactions between body mass and whether a crab was vitellogenic (*t* = −2.15, *p* = .043), between average temperature during a trial and whether a crab was vitellogenic (*t* = −1.99, *p* = .059), and between body mass and the number of missing limbs (*t* = 2.57, *p* = .017). Overall, these predictor variables explained 87% of the variation in metabolic rate in female crabs.

**FIGURE 4:**
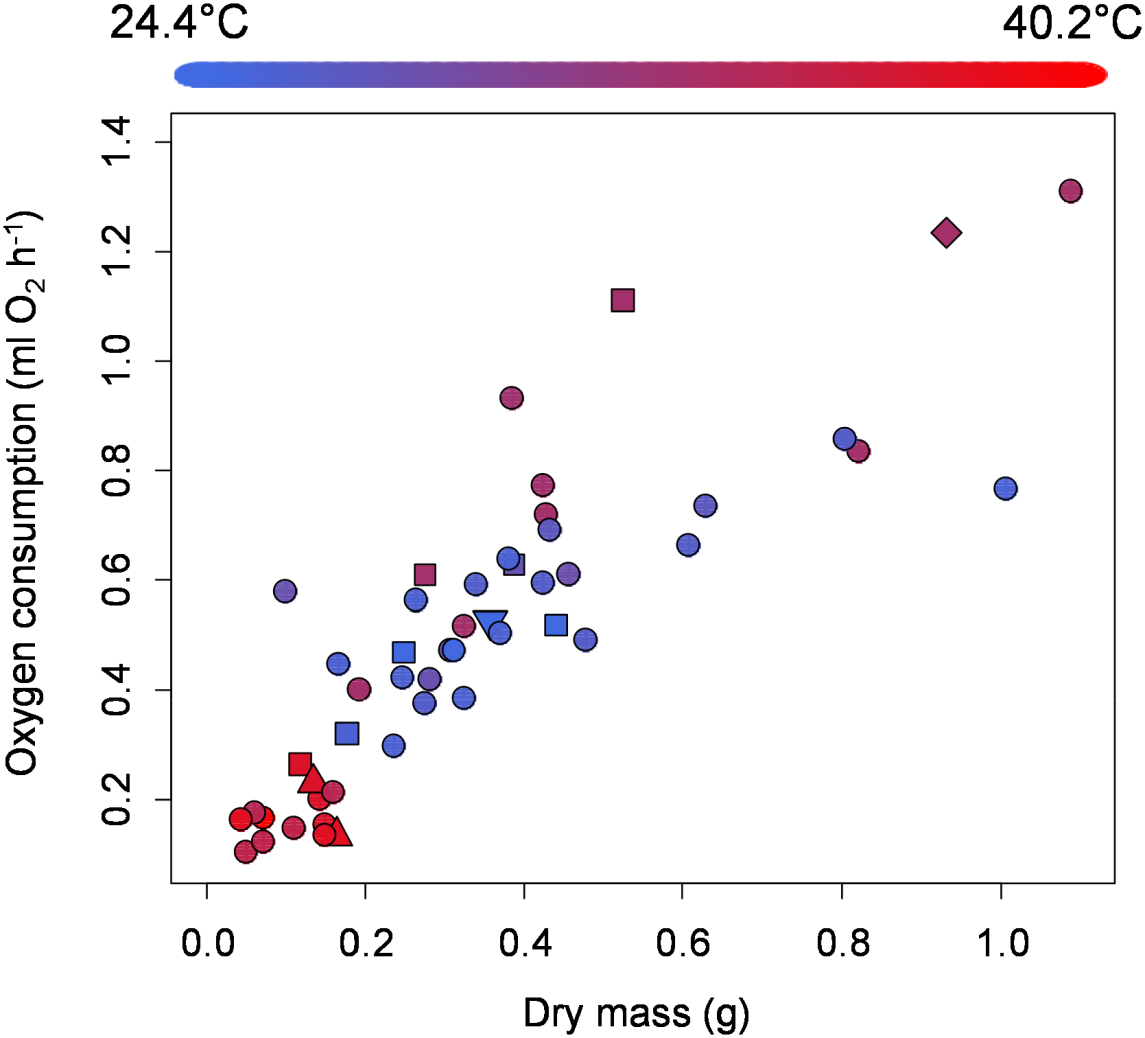
Metabolic rate of male *Armases cinereum* as a function of body mass (*x*‐axis), the number of limbs that were missing (symbol type: circle = 0, square = 1, diamond = 2, triangle = 3, upside down triangle = 4), and the average temperature during experimental trials (symbol colors using the scale shown long the top of the graph).

For male crabs, metabolic rate was influenced by body mass (main effect not significant due to interactions: *t* = −1.06, *p* = .300, Figure 4), by average temperature during the trial (main effect: *t* = −3.93, *p* = .0005, Figure 4), by the number of limbs that were missing (main effect, *t* = 3.30, *p* = .003, Figure 4), and regenerating (main effect *t* = −3.03, *p* = .005). As with females, the variables interacted in complex ways to influence metabolism (mass × temperature interaction *t* = 1.73, *p* = .094; body mass × number limbs missing interaction *t* = −3.46, *p* = .002; temperature × number limbs missing interaction *t* = −2.97, *p* = .006; body mass × number limbs regenerating interaction *t* = 3.80, *p* = .0006; and temperature and the number of limbs regenerating *t* = 2.43, *p* = .021). Overall, these four factors explained 87% of the variation in metabolic rate in male crabs.

## 4 DISCUSSION

Our results showed that metabolic rate increases with size for both males and females, but that rates increase faster with body mass for females than for males. They also indicate that vitellogenic females had higher metabolic rates than non‐vitellogenic females. The metabolic rate of both sexes was also influenced by ambient temperature and by nonlethal injury (limb loss). In addition, for both males and females, these factors interacted with each other in their influence on metabolic rates.

The metabolic rates that we obtained are approximately an order of magnitude higher than those reported in the literature for this species (Full et al., 1985; Teal, 1959), and this may be attributed to various causes. Metabolic rates here were likely elevated compared to previously reported values due to specific dynamic action, or the extra metabolic cost of digestion. As the crabs were dissected, we visually assessed their guts to determine whether they were empty or contained food. We found that approximately 70% of crabs had food in their guts at the time metabolic measurements were taken. In green crabs (*Carcinus maenas*) and in ghost crabs (*Ocypode quadrata*), specific dynamic action increases the metabolic rate 3–5× the rate when the individuals are starved before measurements (Burggren et al., 1993; Robertson et al., 2002). Elevated metabolic rates were also likely caused by relatively high ambient summer temperatures at which measurements were made. For both females and males, temperature was a significant factor in metabolic rate in our results. Temperatures in previous studies were not reported, but historically, metabolic rates have mostly been made at standard conditions (i.e., 25°C), while we measured rates at field conditions that ranged from 24.4 to 40.2°C across trials. Lastly, activity can increase the metabolic rate of the individuals. Though crabs were mostly inactive in the experimental syringes during our trials, activity has been shown to increase the metabolic rate in blue crabs (*Callinectes sapidus*) and ghost crabs (*O. quadrata*) by 5× and 3.5× the resting rate, respectively (Booth & McMahon, 1992; Full et al., 1983).

Regardless of the reason, or combination of reasons, for the elevated metabolic rates measured here, these high metabolic rates suggest that a large portion of consumed energy is expended by *A. cinereum* under normal field conditions. Previous studies noting the high mobility of *A. cinereum* across marsh and forest ecotones have posited the potential importance of this species as a vector for energy subsidies (Hübneṙ et al., 2015). Given the high metabolic rates reported here that are likely indicative of rates under normal daily conditions, further work is required to determine how much energy is consumed daily in the marsh, and the portion of that energy that is expended in metabolic maintenance costs, or that is alternatively available as a potential subsidy, either through fecal deposition or via consumption of crabs by forest predators. The large gut of this species relative to other brachyuran crabs (Griffen & Mosblack, 2011) may enable sufficient consumption that, even with the high metabolic energy expenditure reported here, individuals may still act as vectors to transport a substantial amount of food (energy) from the marsh to the forest ecotone.

As noted above, this question could ideally be addressed using a bioenergetics model. Our data provides a necessary component of a bioenergetics model for *A. cinereum*. Given the widespread distribution and high abundance of this species, understanding its energy budget could aid in understanding the overall energy flow in upper saltmarsh ecosystems, and its quantitative role as a vector of spatial subsidies within this system (Hübneṙ et al., 2015). The majority of data needed for a bioenergetics model for *A. cinereum* are now available (consumption: Ho & Pennings, 2008; Hübneṙ et al., 2015; Kiskaddon, 2016; Kiskaddon et al., 2019; growth: Buck et al., 2003; Zimmer et al., 2004; reproduction: Figueiredo et al., 2008, respiration: this study). Additional data needs for constructing such a model include estimates of assimilation and of the energy content of eggs. Finally, while previous research demonstrates the feeding patterns of *A. cinereum* (Ho & Pennings, 2008; Hübneṙ et al., 2015; Kiskaddon, 2016; Kiskaddon et al., 2019), the amount of food consumed by this species has yet to be quantified. The large gut of this species relative to other brachyuran crabs (Griffen & Mosblack, 2011) suggests that consumption rates may be substantial.

## AUTHOR CONTRIBUTIONS

**Nanette Smith:** Data curation (equal); writing – original draft (lead). **Lars Anderson III:** Data curation (equal); writing – review and editing (equal). **Laura S. Fletcher:** Data curation (equal); writing – review and editing (equal). **Carter K. Stancil:** Data curation (equal); writing – review and editing (equal). **Blaine D. Griffen:** Conceptualization (lead); data curation (equal); formal analysis (lead); funding acquisition (lead); methodology (lead); project administration (lead); visualization (lead); writing – review and editing (lead).

## Data Availability

All data contained in this paper have been deposited in Dryad (https://doi.org/10.5061/dryad.w0vt4b8w2).
